# A pilot feasibility study of a peer-led mindfulness program for medical students

**Published:** 2016-03-31

**Authors:** Marlon Danilewitz, Jacques Bradwejn, Diana Koszycki

**Affiliations:** 1 University of British Columbia, Department of Psychiatry, Vancouver, BC; 2 University of Ottawa, Faculty of Medicine, Ottawa, ON; 3 Institut de recherche de l’hôpital Montfort, Ottawa, ON; 4 University of Ottawa, Faculty of Education, Ottawa, ON

## Abstract

**Background:**

Mindfulness meditation has gained momentum in medical circles for bolstering wellbeing and other facets of professionalism. This study evaluated the feasibility and benefits of a peer-led mindfulness meditation program (MMP) on medical student wellness and professionalism.

**Method:**

Pre-clerkship students were recruited and randomized to the 8-week MMP or wait-list. Feasibility outcomes included ease of recruitment, program attendance and homework compliance. Other outcomes included self-reported psychological distress, empathy, self-compassion, mindfulness, altruism and program satisfaction.

**Results:**

The MMP decreased levels of stress and enhanced mindfulness, self-compassion and altruism from baseline to post-study. Changes were not significant for the wait-list condition. Although satisfaction with the MMP was high compliance was suboptimal.

**Conclusions:**

A peer-led MMP is feasible and may be a promising approach to enhance medical student wellbeing. Further research is needed to explore strategies to improve program compliance in this student population.

## Introduction

Medical school is a stressful and challenging time for students. Studies have demonstrated that rates of psychological distress are high among students.^1^ Similarly, risk for depression is elevated in medical students and increases with years of study.^2^ Increased levels of depression have been linked to other poor outcomes, including dropout and problematic alcohol consumption.^2^ Moreover, physician distress and depression have been shown to correlate with decreased quality of patient care, reduced empathy and increased incidence of medical error.^3^ Despite high levels of psychological distress, mental health service use among medical students is low. The lack of free time, perception of academic jeopardy, concern for confidentiality, stigma and cost are all prominent obstacles that discourage medical students from seeking mental health support.^2^

Student wellness has become a priority in many medical schools. Although a number of initiatives have been developed to foster positive mental health in medical students, the effectiveness of these programs has not been well researched. One intervention that has gained momentum in medical circles for its effectiveness in bolstering wellbeing and other facets of professionalism is mindfulness meditation.^4^ Mindfulness meditation is an ancient Buddhist practice that can be learned independently of any spiritual or cultural tradition.^5^ It is a type of mind training that teaches participants to be more aware, to relate differently to thoughts, feelings and sensations, and to express greater moment-to-moment awareness. These attributes help participants cultivate a more adaptive and healthier response patterns to stress.

While mindfulness-based programs have been evaluated in medical student populations, no previous investigations have incorporated medical students as part of the core research development team. Medical students, a group with an otherwise vibrant and powerful voice, have been silent regarding the domain of their own wellbeing. Previous research has also documented that medical students are reluctant to seek mental health support from professionals.^6^ Our study reflects the work and efforts of a grassroots initiative started, conducted and researched by a medical student. Involving medical students more intimately in their own education and wellness may in fact be the best approach to address many of the aforementioned barriers of seeking support. Participating in a peer-led program may be perceived as being less stigmatizing than participating in programs led exclusively by mental health professionals or medical faculty. Participants may also better relate to peer leaders given their shared experience, and may feel more openness to sharing personal information in this judgment-free setting.

The primary aim of this pilot study was to assess the overall interest and ability of pre-clerkship medical students to participate in an 8-week peer-led mindfulness meditation program (MMP). A secondary aim was to obtain preliminary data on the efficacy of the program in improving medical student wellness, mindfulness and professionalism skills.

## Methods

### Study design

The study used a randomized waitlist (WL) control design. Participants assigned to the WL received the intervention following the 8-week wait period.

### Participants

Participants were pre-clerkship medical students at the University of Ottawa who were recruited at the beginning of September 2012. All first and second year medical students (n=300) received an email inviting them to participate in the study. The first 30 students who responded to the recruitment announcement were included in the pilot study. Students who consented to participate received medical school performance record (MSPR) credit through the Wellness Student Interest Group. The study received ethics approval from the Ottawa Health Science Network Research Ethics Board.

### Intervention

The MMP was an adapted version of the mindfulness based stress reduction (MBSR) program developed by Kabat-Zinn.^7^ MBSR is the most well-established secular mindfulness training program, and it has been evaluated in diverse populations including health care professionals.^4^ The MMP used in our study was specifically designed for medical students and informed by the curriculum developed by Epstein and colleagues.^8^ Session themes addressed core issues that face medical students including work life balance, dealing with patient suffering, being mindful in clinical interactions, dealing with the need to be perfect, and self-acceptance (Table 1). The program was led by a medical student peer with professional training in MBSR. A psychologist with expertise and training in mindfulness meditation co-facilitated the program. The intervention encompassed 8, 1–1.5 hour weekly sessions. Participants were provided recordings of the meditation exercises to practice each day at home. Weekly homework forms were completed to assess home practice between formal sessions.

### Outcomes measures

Our feasibility outcomes included ease of recruitment, program retention and homework compliance. We considered the study feasible if 75% of participants completed four or more sessions and if compliance with homework completion was 70% or higher. Efficacy outcomes included the following self-report scales: the Depression Anxiety and Stress Scale,^9^ a 42–item scale that assesses three negative emotional states of tension/stress, anxiety and depression over the past week; the Jefferson Scale of Physician Empathy -Student Version,^10^ a 20-item questionnaire assessing health care professional empathy in patient care settings; The Five Facets of Mindfulness Questionnaire (FFMQ),^11^ a 39-item questionnaire exploring mindfulness in daily life. The five facets are observing, describing, acting with awareness, non-judging of inner experience and non-reactivity to inner experience; the Self-Compassion Scale (SCS),^12^ a 26-item self-report scale that captures how respondents perceive their actions toward themselves in difficult times; and the Adapted Altruism Scale,^13^ a 14-item scale that assesses altruistic behaviors. These scales were completed at baseline and at the end of the program or wait period.

A five-point Likert scale, ranging from Strongly Disagree (1) to Strongly Agree (5), was used to assess student satisfaction with the MMP and perceived benefit on levels of academic and clinical performance. All participants who completed the mindfulness program, including the WL participants who received the intervention after the wait period, completed the satisfaction questionnaire.

### Statistical methods

Data were analyzed with SPSS Version 21. Main analyses for this study included calculation of feasibility outcomes using descriptive statistics. Efficacy analysis was performed on the intent-to-treat sample. For students with missing post-study assessments, we carried forward their baseline scores as an approach to imputing missing data (i.e. last observation carried forward - LOCF). Paired sample t-tests were used to evaluate pre- to post-study changes in outcome within each group. Between-group comparisons were assessed with analysis of covariance (ANCOVA), with the baseline score of each measure used as covariate. Within- and between-group effect sizes (Cohen’s d) were calculated to examine the magnitude of effect of the MMP. Calculation of within-group effect sizes accounted for correlations between repeated measures. According to convention, a small effect is considered to be .20, a medium effect .50 and a large effect .80. Significance was set at p<0.05, two-tailed tests.

## Results

We recruited our target sample of 30 pre-clerkship students within the first two weeks of active recruitment. Our sample was predominately female (*n*=22, 73.3%) and equally composed of first (*n*=14) and second (*n*=16) year students. The average number of sessions attended was 3.9 out of 8 sessions. Nine (60%) participants completed four or more sessions. Among participants who dropped out early (*n*=6; 40%) one did not start the program, 4 dropped out after session 2 and one dropped out after session 3. Regarding homework compliance, only 4 participants reported a regular meditation practice. Post-study assessments were completed by 80% (*n*=13) of MMP and 60% (*n*=9) of WL participants.

Table 2 displays descriptive statistics for the efficacy outcomes and within- and between-group effect sizes for the intent-to-treat sample. Paired *t*-tests revealed that participants assigned to the MMP showed significant pre- to post-test changes in levels of stress (*p*=.019), self-compassion (*p*=.024), altruism (*p*=.033) and two facets of mindfulness: describe (*p*=.05) and non-react (*p*=.034), with the magnitude (effect size) of change ranging from small to medium. Pre- to post-test changes were not significant for the WL. Between group comparisons revealed that post-test scores on the FFMQ-observe subscale were significantly higher for the MMP versus WL (*p*=.026), with a moderate effect size.

All participants who received the MMP reported a high level of satisfaction with the program. 88% reported that the program had a positive impact on their academic performance and 69% reported a positive impact of the program on their clinical performance.

## Discussion

To our knowledge this is one of the first studies to evaluate the feasibility and potential benefits of a peer-led MMP for medical students. Although the sample size is small and compliance with the program was suboptimal, results are nevertheless encouraging and suggest that a peer-led MMP may have a positive impact on student wellbeing, mindfulness, self-compassion and aspects of professionalism.

There has been a shift in medical education over the recent past from top-down teaching models towards peer teaching.^14^ In keeping with this shift, there may be several potential benefits to offering mindfulness meditation programs for medical students as a peer-led program. First, our success with recruitment and the high rate of program satisfaction suggest that a peer-led mindfulness-based wellness program is acceptable to medical students. The strong student interest in the current study subsequently spurred additional wellness related initiatives, including a student/faculty-working group for integrating mindfulness into the formal curriculum in our medical school program.

Training in mindfulness demands a considerable time-investment from participants and, as noted above, the rate of program completion in this study was lower than expected with 40% of participants failing to complete at least 4 of the 8 weekly lunch-time sessions. Lamothe and colleagues^15^ reported that attrition rates amongst health care professionals in mindfulness programs range between 11 to 37%, with one study reporting a 50% attrition rate.^16^ Our study highlights the challenges of offering an 8-week mindfulness program to medical students. Potential difficulties that may influence program adherence among these students include multiple demands on their time and lack control over their own schedule: medical students are obligated to attend both clinical and educational programming, which often change based on the rotation and stage of learning they are in. Moreover, their schedules are subject to change in order to accommodate examinations. Future research to explore ways of improving program attendance would be of benefit, including evaluating the ideal number and length of MMP sessions, developing strategies to encourage students to remain in the program, and identifying factors that predict attrition.

Similarly, participants reported difficulty engaging in daily mindfulness home practice, although they were more likely to practice yoga or informal meditation practices (i.e., mindfulness in daily activities). The level of homework compliance observed in this study is consistent with other reports that mindfulness home practice among participants in mindfulness-based interventions is low.^17^,^18^ In a study of nursing students, only 12% established a daily meditation practice,^19^ with mindful yoga being the most frequent activity among those who reported home practice. Similarly, in a study of first year medical students who attended a modified 8-week MBSR course, adherence to the home practice was low, despite students being enthusiastic about the program.^20^ Improving compliance with mediation practice and determining the optimal “dose” and preferred type of practice (i.e., sitting meditation versus mindful yoga) is clearly an important issue to address as establishing a daily formal meditation practice is necessary to effect change in one’s capacity to self-regulate emotions and levels of mindfulness.^21^

Because sessions were held during the day, third and fourth year medical students were automatically excluded from participating in this study. An interesting avenue for future research would be to examine the impact of an online mindfulness program that would allow greater participation of third and fourth year students, and allow greater ease for first and second year medical students to engage in ongoing mindfulness training at their own pace. Online mindfulness and psychological interventions have been shown to be similarly effective as face-to-face equivalents, while also being a more feasible option given the reduced cost and logistical requirements.^22^ Integrating elements of mindfulness training into the formal curriculum for pre-clerkship and clerkship medical students is another potential avenue for addressing the issues of feasibility and attendance, which is currently being explored at our faculty and in other medical schools.^23^ Whether students actually adopt a regular meditation practice following formal lectures/tutorials on mindfulness is another important question to investigate.

There are study limitations to note. First, the small sample size limits the generalizability of findings to other medical students at the University of Ottawa, and reduces the power to detect differences between the MMP and WL condition on most outcomes. Second, the completion rate of post-study questionnaires was lower in the WL than MMP condition, and the use of the last observation carried forward method to impute missing data may be problematic here. Although a linear mixed model would have been ideal as this approach uses all time points without the need to use imputation procedures, our sample size was too small for this analytic approach. Third, the sample comprised predominantly female medical students, although this is in keeping with gender ratios reported in the literature. In a review of 39 mindfulness studies involving health care provider populations 81% of participants were female.^16^ Fourth, it would have been more rigorous to have included an active control group which would have allowed us to discern between the effects of the mindfulness training itself and any benefits incurred simply from the experience of being in collegial and supportive environment. Finally, students who volunteered to participate in the study may represent a self-selected sample that was more receptive to mindfulness training or in greater need of a stress reduction program.

### Conclusion

Although these preliminary findings must be viewed with caution, results suggest an 8-week peer-led mindfulness based intervention is acceptable to pre-clerkship medical students and may improve well-being and aspects of professionalism for those who express interest in such an intervention. Further research on the benefits of a peer-led mindfulness program is warranted.

## Figures and Tables

**Table 1:** Module themes

Module	Themes	Meditation Practice
1	Introduction to Mindfulness	Body Scan
	Mindfulness and Clinical Medicine	Raisin Exercise
2	Barriers to Mindfulness in Medical School	Body Scan
	Interacting With Our Own Suffering and Patient Suffering	
3	Being Mindful in Everyday Life and Medical School	Awareness of Breath
	The Five Skillful Habits of Mindfulness	
4	Dealing with Stress	Awareness of Breath
	Emotional Appraisal and Learning How to Respond and Not React	
5	Relating to Our Sensations	Yoga
	Self Acceptance: Dealing with Perfectionism	
6	Relating to Our Judging Mind	Walking Meditation
7	Loving Kindness	Loving Kindness Meditation
	Being Compassionate to Ourselves and Our Patients	
8	Integrating Mindfulness into Our Lives	Sitting Meditation
	Moving Beyond the Program	

**Table 2:** Means (SD) and within- and between-group effect sizes for the intent-to-treat sample

	Mean (SD)		Within group effect sizes	Between group effect sizes
	Pre	Post		
Stress				
MMP	17.3±7.66	12.3±6.6	.70	.33
WL	15.8±9.8	14.7±7.9	.12	
Depression				
MMP	7.6±5.2	5.4±3.9	.48	.02
WL	6.0±9.1	5.5±6.0	.07	
Anxiety				
MMP	9.9±6.3	7.9±6.3	.31	.50
WL	6.3±8.4	5.2±4.2	.10	
Observe				
MMP	27.3±5.7	28.7±5.8	.24	.63
WL	27.1±5.6	25.7±4.9	.25	
Describe				
MMP	28.3±6.5	30.2±8.1	.24	.63
WL	25.8±5.2	26.0±4.9	.04	
Nonreact				
MMP	21.6±4.7	23.2±5.3	.31	.21
WL	21.5±4.6	22.2±3.8	.20	
Nonjudge				
MMP	24.7±8.0	27.3±7.5	.34	.04
WL	26.2±5.7	27.0±6.5	.13	
Awareness				
MMP	23.9±5.2	25.5±4.5	.33	.08
WL	24.6±5.7	25.1±5.1	.10	
Empathy				
MMP	117.1±8.1	118.8±9.4	.19	.21
WL	118.1±9.4	116.6±11.6	.14	
Self Compassion				
MMP	79.8±15.6	86.0±18.4	.35	.38
WL	76.3±17.1	79.7±14.8	.21	
Altruism				
MMP	39.5±6.6	42.6±6.6	.46	.65
WL	37.6±7.4	37.5±9.0	.003	
